# The Association between Circulating MicroRNA Levels and Coronary Endothelial Function

**DOI:** 10.1371/journal.pone.0109650

**Published:** 2014-10-13

**Authors:** R. Jay Widmer, Woo-Young Chung, Joerg Herrmann, Kyra L. Jordan, Lilach O. Lerman, Amir Lerman

**Affiliations:** 1 Division of Cardiovascular Diseases, Department of Internal Medicine, Mayo Clinic and College of Medicine, Rochester, Minnesota, United States of America; 2 Division of Nephrology and Hypertension, Department of Internal Medicine, Mayo Clinic and College of Medicine, Rochester, Minnesota, United States of America; University of Nebraska Medical Center, United States of America

## Abstract

Human microRNAs (miRs) have been implicated in human diseases presumably through the downregulation and silencing of targeted genes via post-translational modifications. However, their role in the early stage of coronary atherosclerosis is not known. The aim of this study was to test the hypothesis that patients with early atherosclerosis and coronary endothelial dysfunction (CED) have alterations in transcoronary miR gradients. Patients underwent coronary angiography and endothelial function testing in the cardiac catheterization laboratory. Patients were divided into abnormal (n = 26) and normal (n = 22) microvascular coronary endothelial function based on intracoronary response to infused acetylcholine measured as a percent change in coronary blood flow (CBF) and arterial diameter. Blood samples were obtained simultaneously from the aorta and coronary sinus at the time of catheterization for RNA isolation, and miR subsequently assessed. Baseline characteristics were similar in both groups. Patients with microvascular CED displayed transcoronary gradients significantly elevated in miR-92a and miR-133 normalized to C-elegans-39 miR. Percent change in CBF and the transcoronary gradient of miR-133 displayed a significant inverse correlation (r^2^ = 0.11, p = 0.03). Thus, we present novel data whereupon selected miRs demonstrate elevated transcoronary gradients in patients with microvascular CED. The current findings support further studies on the mechanistic role of miRs in coronary atherosclerosis and in humans.

## Introduction

Human microRNAs (miRs) comprise a large collection of small non-coding RNAs (17–22 nucleotides) responsible for modification of post-translational gene expression by promoting degradation or repressing translation of coding mRNAs [1]. These diminutive nucleotides present a very small fraction of tissue and blood content (approximately 0.5%) [2], which is altered in patients with malignancy [3], coronary heart disease (CHD) [4], [5], and acute kidney injury [6]. Initial work highlighted the cardiovascular importance of miRs as decreased levels of the miR processing enzyme, Dicer, causes dysfunctional angiogenesis and cardiovascular development [7]. Several miRs have been implicated in regulating cardiovascular structure and function. miR-17, miR-92a, and miR-126 have typically been described in an endothelial-related cluster, whereby miR-17 and miR-92a have typically been considered as attenuating endothelial function or angiogenesis [8], [9], and miR-126 thought to be atheroprotective [10], [11] as well as pro-angiogenic [12]. miR-34 is upregulated in the bone marrow derived mononuclear cells in patients with ACS [13]. miR-133 and miR-208 converge around structures pertaining to myocardial tissue albeit with opposite effects on cardiac hypertrophy, as miR-133 yields both anti-fibrotic and anti-hypertrophic effects [14], whereas overexpression of miR-208 is pro-hypertrophic [15]. miR-21 has recently been found to be protective in an ischemic-reperfusion model through anti-apoptotic means [16]. miR-181 is thought to be atheroprotective by inhibiting LDL oxidation [17], and miR-221 and miR-221 have atherogenic potential in smooth muscle cells [18] with anti-atherogenic potential along the endothelium [19]. Finally, miR-145 is thought to be critical in myocardin-induced smooth muscle cell differentiation [20], and miR-155 has been associated with macrophage activation and increased atherosclerotic burden [21].

A recent study uncovered an association between a series of miRs specific to the myocardium (miR-133a, miR-208a), the vasculature (miR-17, miR-92a, miR-126), inflammatory cells (miR-145), smooth muscle and (miR-155) and the presence of stable CHD [22]. In particular, an absolute and relative decrease in endothelial-related miRs (miR-17, miR-92a, miR-126), smooth muscle miR (miR-145), and inflammatory cell-mediated miR (miR-155) with a converse increase in miR-133a and miR-208a (myocardial-associated miRs) was observed in patients with stable CHD [22]. On the other hand, in patients with active acute coronary syndromes (ACS) miR-133a and miR-208a (myocardial miRs), miR-126 and miR-92a (endothelial), and miR-155 (inflammatory cell) levels were all found to be increased in aortic blood samples (miR-133a, miR-208a also increased in coronary sinus samples), with increased transcoronary gradient of miR-133 and trends toward negative gradients of miR-92a and miR-126 [23].

Coronary endothelial dysfunction (CED) is regarded as the earliest stage of coronary atherosclerosis, responsible for increased cardiovascular morbidity and mortality [24], [25], [26], [27]. It can be detected via an inappropriate vasomotor response to physiologic or pharmacologic stress such as the endothelium-dependent vasodilation to acetylcholine (Ach) [24], [25]. Once atheromatous plaques develop, they are noted to be lipid-rich with oxidized LDL (Ox-LDL), inflammatory cells such as macrophages, and calcified necrotic cores all initiating and feeding a cycle of increased reactive oxygen species (ROS), inflammation, and apoptosis, which might progress to cause myocardial infarction [28]. As such, CED serves as an important barometer for cardiovascular health. However, it is unclear if endothelial, myocardial, or inflammatory-related miRs are altered within the coronary circulation in patients with early atherosclerosis.

The current study was therefore designed to test the hypothesis that patients with CED have variations in the transcoronary gradients of circulating coronary miRs.

## Materials and Methods

### Human subjects and recruitment

The Institutional Review Board of Mayo Foundation approved the study and all study subjects provided written, informed consent. The study was conducted in a manner similar to prior work in our lab [29], [30], [31]. A total of 48 patients undergoing coronary angiography and coronary endothelial function testing for clinical indications (referred to the catheterization laboratory for recurrent angina with or without a positive stress test) who met the criteria outlined below for inclusion into the two study groups were recruited. Patients with ACS (unstable angina or acute myocardial infarction), heart failure (ejection fraction <50%), or severe renal or liver disease were excluded. The groups were classified as having normal microvascular or macrovascular endothelial function as assessed by intra-coronary Ach challenge (see below), *n* = 48; or patients with CED, defined by the absence of significant structural coronary lesions on angiography but with abnormal microvascular or macrovascular endothelial function, *n* = 26 and *n* = 9, respectively.

### Study protocol/Hemodynamic measurements

Patients underwent a diagnostic coronary angiography using standard clinical protocols [29], [30], [31], [32]. Patients who met the inclusion criteria were studied and a 5 F multipurpose Amplatz left catheter was placed into the coronary sinus under radiographic guidance via a 7 F femoral vein access after the administration of 5000 units of heparin. The position of the catheter within the coronary sinus was verified by pressure transduction, contrast injection, and oxygen saturation [33], [34]. Blood (20 mL) was drawn simultaneously from both the aortic and coronary venus sinus (CVS) catheters and the coronary guide at the left main coronary artery along with peripheral blood samples used for additional biochemical analyses [33], [34]. All subjects underwent assessment of endothelium-dependent coronary vasoreactivity, as previously described [30], [31]. In brief, 5000 units of heparin were given intravenously and a Doppler guidewire (Flowire, Volcano Inc.) within a coronary-infusion catheter (Ultrafuse, SciMed Life System) positioned into the mid-portion of the left anterior descending coronary artery. Ach at increasing concentrations (10^−6^ to 10^−4^ M) was infused into the left anterior descending coronary artery to assess endothelium-dependent vasoreactivity. Hemodynamic data, Doppler measurements, and a coronary angiogram were obtained after each infusion. An independent investigator measured coronary artery diameter (CAD) in the segment 5 mm distal to the tip of the Doppler wire using a computer-based image analysis system, as well as 5 mm proximal and distal to the velocity probe. Average peak velocity (APV) derived from the Doppler flow velocity spectra and coronary blood flow (CBF) was determined as π(coronary artery diameter/2)^2^× (APV/2). As previously described, microvascular endothelial dysfunction was defined as an increase in CBF of <50% and epicardial endothelial dysfunction as a decrease in epicardial coronary artery diameter of more than 20% in response to the maximal dose of ACh (10^−4^ M) [35]. The study population was stratified by both coronary microvascular and epicardial vessel function. Endothelium-independent microvascular function was determined by the coronary flow reserve (CFR), which is the ratio of the APV at maximal hyperemia [induced by intracoronary adenosine (24–60 µg)] to the APV at baseline [36].

### MicroRNA Quantification

MicroRNA levels were determined in a fashion similar to that described previously [22], [23]. Briefly, total RNA was extracted and isolated from the serum using a commercially available kit (Qiagen) according to the manufacturer's instructions. Reverse transcription was then performed using the previously obtained RNA (TaqMan miRNA reverse transcription kit, Invitrogen). Polymerase chain reaction was then undertaken with a reaction volume of 20 µl and the TaqMAN miRNA assay kit according to manufacturer's instructions for each miR (Invitrogen). miRs studied included miR-17, miR-92a, miR-126, miR-34, miR-181b, miR-221, miR-222 (endothelium related), miR-208, 133 (myocardium related), miR-21, miR-145 (vascular smooth muscle related), and miR-155 (Inflammatory cell related). miR quantification was performed using the delta-Cp technique (Roche) with c-elegans-39 serving as the internal standard [37]. Standard curves were obtained for miR-92a, miR-126, miR-181b, miR-221, and miR-222. Absolute Cp counts were used to calculate delta-Cp relative quantifications for both aortic and CVS samples. Transcoronary gradients were calculated as the difference between relative, delta-Cp quantifications between the CVS and aortic samples (CVS-Ao).

### Biochemical assays

Biochemical assays of systemic surrogate markers of CHD were performed as previously described [29]. Serum lipids were measured using enzymatic colorimetry and LDL cholesterol calculated from these parameters. Hemoglobin A1c was measured using ion-exchange high performance chromatography (BIO-RAD Variant II Turbo Hemoglobin A1c program, Hercules, CA, USA). Free insulin levels were measured by an automated chemiluminescent immunoenzymatic assay (ACCESS, Beckman-Coulter Inc., Fullerton, CA, USA). High sensitivity C-reactive protein (hs-CRP) levels were measured using a latex particle-enhanced immunoturbidemetric assay on a Hitachi 912 automated analyser. Serum creatinine was measured as described before [36].

### Statistical analyses

Data was expressed as mean ±SD where normally distributed. Baseline comparisons were made by way of student's *t*-test for continuous data or Fischer's Exact Comparison for binary measures. Comparison of different groups was performed by one-way ANOVA or Spearman's correlation with the ANOVA tests followed by post hoc tests for parametric and nonparametric distribution. Comparisons between the two groups were made by Student's *t*-test for normally distributed data or by Wilcoxon signed rank test for non-normally distributed data. A value of *P*<0.05 was considered significant. Correlations between specific miRs and systemic levels or surrogate markers for CHD were analyzed by multivariate analysis and reported as Spearman's correlation coefficients.

## Results

Among patients who underwent coronary endothelial testing (n = 48), there were few differences in baseline data between those with and without microvascular endothelial dysfunction (Tables 1 and 2) including increased prevalence of hyperlipidemia and baseline vitamin B12 levels in patients with normal endothelial function.

**Table 1:** Baseline characteristics of participants divided into groups based on microvascular endothelial function.

	Normal Microvascular Endothelial Function (N = 22)	Abnormal Microvascular Endothelial Function (N = 26)	p-value
**Male**	7/22 (32%)	9/26 (35%)	0.84
**Age (years)**	53.8 (+2.3)	52.1 (+2.5)	0.62
**Currently Smoking**	0/18 (0%)	1/18 (6%)	0.5
**BMI (kg/m^2^)**	30.5 (+1.6)	30.6 (+1.6)	0.96
**Family History of CVD**	10/18 (56%)	8/17 (47%)	0.61
**Diabetes Mellitus type 2**	1/19 (5%)	2/18 (11%)	0.51
**Hypertension**	11/19 (58%)	8/17 (47%)	0.51
**Hyperlipidemia**	16/19 (84%)	8/18 (44%)	0.01*
**Vascular Disease**	0/18 (0%)	0/18 (0%)	0.99
**% Change CBF to ACh**	+143.8−+13.7%	−4.7−+12.6%	<0.0001*
**% Change CAD to ACh**	−3.6−+3.0%	−12.8−+2.8%	0.03
**Coronary Flow Reserve**	3.2−+0.17	3.0−+0.16	0.34
**Aspirin Rx**	11/20 (55%)	13/17 (76%)	0.17
**Antihypertensive Rx**	11/20 (55%)	10/17 (59%)	0.86
**Lipid-Lowering Rx**	10/20 (50%)	9/17 (53%)	0.82

Endothelial dysfunction was described as a percent increase in blood flow below 50% in response to maximum acetylecholine. Most of the general demographics were not statistically significant between the groups with the exception of a higher number of diabetics in the normal microvascular function group.

**Table 2:** Surrogate markers for coronary heart disease (CHD) shown divided between those with endothelial dysfunction and those without.

Marker	Normal Microvascular Endothelial Function	Abnormal Microvascular Endothelial Function	p-value
**Hemoglobin** (x10^12^/L)	13.43−+1.56	13.65−+1.06	0.62
**Hematocrit** (%)	41.2−+1	40.5−+1	0.53
**Leukocytes** (x10^9^/L)	6.81−+2.96	6.11−+1.73	0.53
**Monocytes** (x10^9^/L)	0.50−+0.13	0.51−+0.16	0.79
**Lymphocytes** (x10^9^/L)	1.74−+0.78	1.93−+0.61	0.42
**Platelets** (x10^9^/L)	225.6−+53.3	224.0−+40.4	0.92
**Total Cholesterol** (mg/dL)	207.1−+53.8	183.1−+51.3	0.18
**LDL-Cholesterol** (mg/dL)	122.6−+40.7	101.9−+39.9	0.13
**HDL-Cholesterol** (mg/dL)	52.2−+14.1	57.3−+19.4	0.36
**Triglycerides** (mg/dL)	156.8−+108.6	126.9−+58.8	0.31
**Glucose** (mg/dL)	105.1−+16.7	92.9−+25.8	0.09
**Insulin** (micro IU/ml)	12.5−+7.9	14.0−+9.5	0.64
**BNP** (pg/mL)	86.6−+78.4	181.2−+220.5	0.12
**Hs-CRP** (mg/L)	4.1−+4.8	4.0−+5.6	0.97
**Homycystein** (µmol/L)	7.1−+1.6	8.0−+2.7	0.24
**Vitamin B12** (ng/L)	531.8−+273.8	357.4−+122.8	0.02*

Only vitamin B12 levels were statistically different.

Mean aortic miR levels were significantly reduced, after normalization using the delta-CP method, in miR-92a (p = 0.02), miR-126 (p = 0.03), miR-133 (p = 0.03), and miR-155 (p = 0.003). Mean miR levels of miR-126 (p = 0.03) and miR-155 (p = 0.01) from CVS samples were reduced throughout all samples in patients with microvascular CED, yet only significant reduction in miR-155 persisted after relative correction to C-elegans-39 (p = 0.01). Ct values for the C-elegans spike in control were 30.2+4.8.

**Figure 1:**
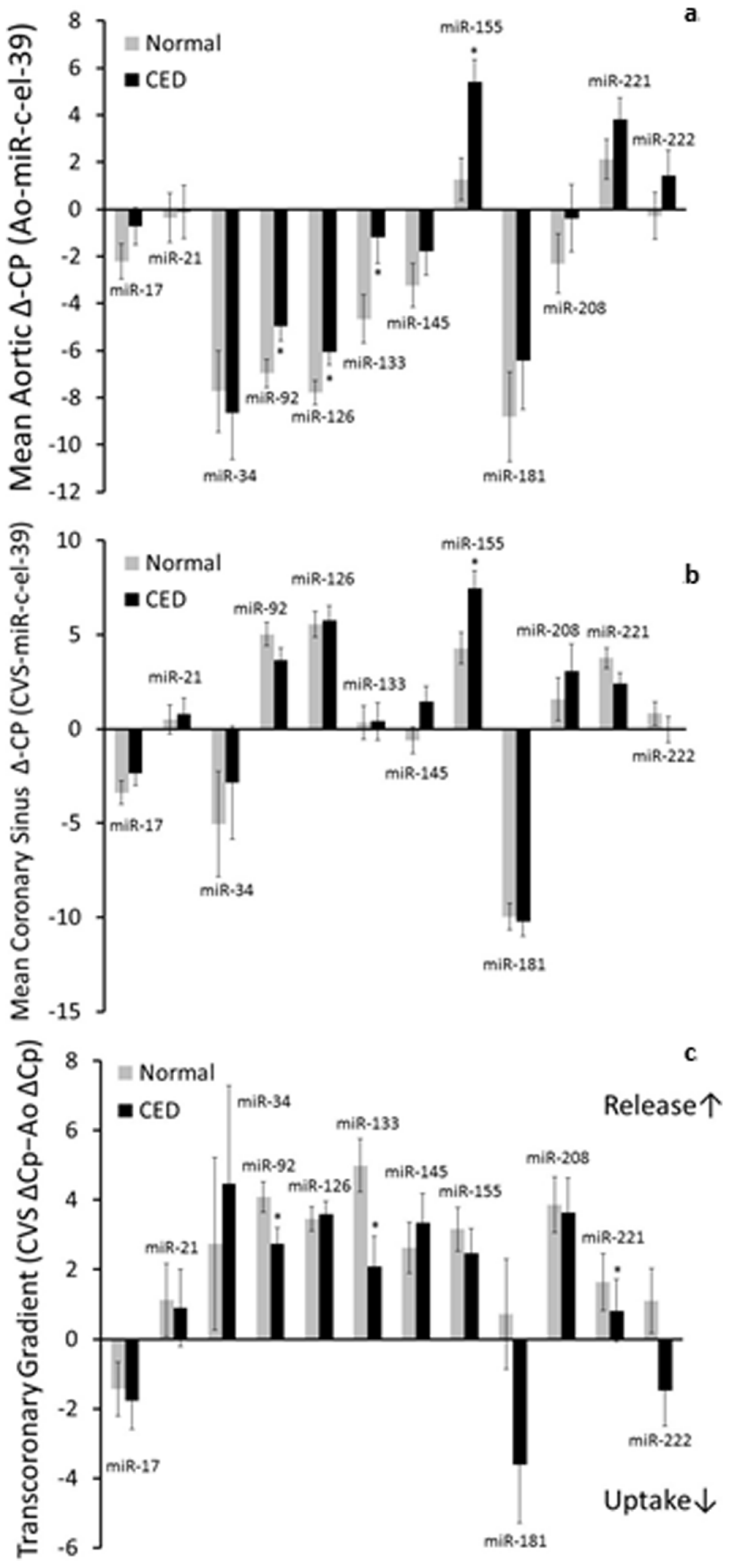
Transcoronary gradients normalized using the delta Cp method, in patients with normal coronary endothelial function and those with CED with regard to endothelial-related miRs (A), myocardial-related miRs (B), and vascular/inflammatory miRs (C). The transcoronary gradients of miR-92a (A) and miR-133 (B) were significantly elevated in patients with CED compared to those with normal coronary endothelial function (*, p<0.05).

**Figure 2:**
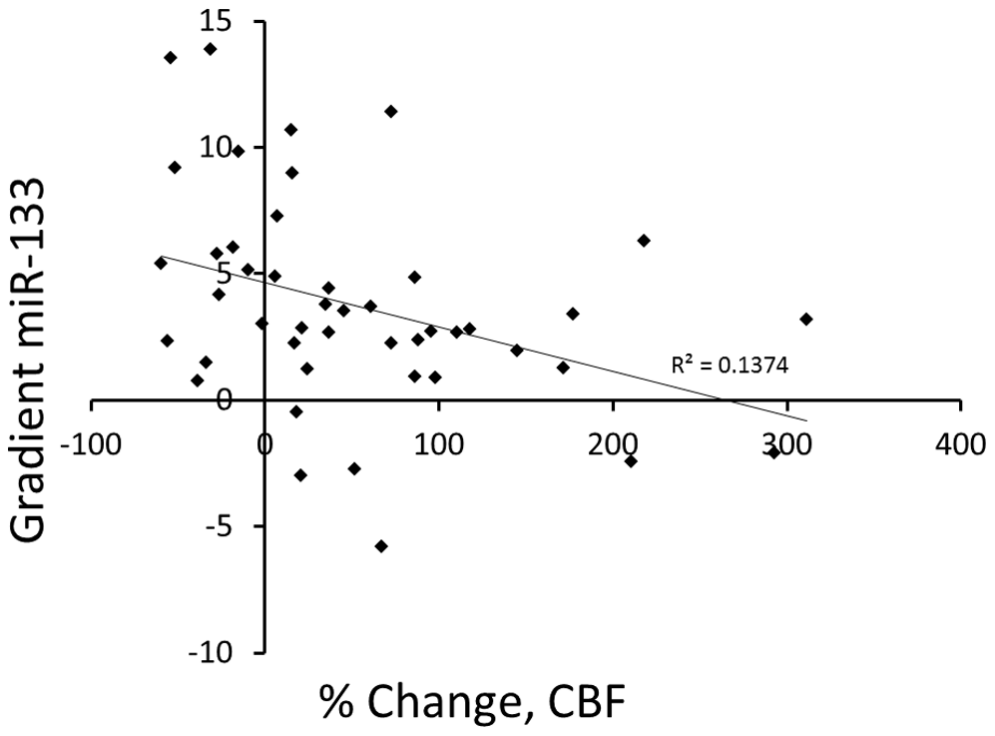
Transcoronary gradient of miR-133 versus the percent change in microcirculatory blood flow showing an inverse correlation (r^2^ = 0.11, p = 0.03).

Transcoronary gradients were positive for all miRs analyzed with the exception of miR17. Transcoronary gradients of miR-92a (p = 0.04), miR-133 (p = 0.02), and miR-221 (p = 0.02) were significantly elevated in patients with microvascular CED compared to patients with normal coronary microvascular endothelial function (Figure 1c). A significant inverse correlation between percent change in CBF and transcoronary gradient of miR-133 (r^2^ = 0.11, p = 0.03; Figure 2) indicated that the gradient reduced with improving endothelial function. Multivariate analysis (Spearman's correlation) revealed a few moderate correlations between certain miRs aortic (Table 3), coronary sinus (Table 4), and transcoronary gradients and surrogate markers for CHD found in serum blood draws such as hemoglobin, leukocytes, platelets, total cholesterol, LDL-cholesterol, triglycerides, hs-CRP, and vitamin B12 (miR-21 (p = 0.02), miR-92a (p = 0.02), miR-126 (p = 0.02), miR-133 (p = 0.03), and miR-155 (p = 0.003); (Table 5).

**Table 3:** Associations between aortic miR levels and surrogate markers of cardiovascular disease.

Marker	miR-17	miR-21	miR-34	miR-92	miR-126	miR-133	miR-145	miR-155	miR-181	miR-208	miR-221	miR-222	
**Hemoglobin**	−0.0823	−0.3291	−0.1390	0.0125	−0.1316	0.0563	0.0858	−0.1005	−0.0149	0.0247	−0.1858	−0.2761	
**Leukocytes**	0.1679	0.1074	0.0688	0.0556	0.2045	0.1862	0.2081	0.3034	0.0163	0.0572	0.0113	−0.0046	
**Monocytes**	−0.0383	−0.0882	0.1006	−0.0783	0.2120	0.2885	0.1914	0.2834	0.0830	−0.1431	0.0363	0.0601	
**Lymphocytes**	0.0650	0.0011	0.2054	0.1144	0.1597	0.3864*	0.1037	0.2681	−0.0158	0.1317	0.0094	−0.0580	
**Platelets**	0.2664	0.3423*	0.4934*	−0.2914	−0.0934	−0.1299	0.0327	0.0560	01881	0.1626	0.3920*	0.4368*	
**Total Cholesterol**	0.1032	0.0864	0.2262	0.3293*	0.4013*	0.3190	0.1815	0.0521	0.3595*	0.2683	0.2007	0.0471	
**LDL-Cholesterol**	0.0831	0.1528	0.3077	0.2193	0.4289*	0.2159	0.1007	0.0345	0.4513*	0.2844	0.2704	0.1226	
**HDL-Cholesterol**	0.1738	−0.0769	0.2585	0.0715	−0.1033	−0.0792	0.0880	−0.1661	0.1405	0.1034	−0.0475	−0.0601	
**Triglycerides**	−0.0308	−0.1031	−0.1639	0.2167	0.1633	0.3603*	0.0132	0.0775	0.1422	0.1380	0.1267	0.0205	
**Glucose**	−0.0184	0.0579	−0.0273	0.0515	0.1261	0.1169	−0.0795	0.0930	0.2181	0.0630	0.0729	0.0263	
**HbA1c**	−0.016	0.0963	0.2757	−0.2632	0.0346	0.0000	−0.0544	0.1972	−0.0223	−0.0997	0.0837	0.1163	
**Insulin**	−0.0984	0.3552*	0.1177	−0.1312	−0.1430	0.0541	−0.2134	−0.0007	0.0104	0.0275	0.0545	−0.0353	
**BNP**	−0.1237	−0.1926	−0.4817*	0.0443	−0.3683*	−0.0194	−0.2677	−0.1084	−0.2625	−0.0578	−0.3090	−0.0763	
**hs-CRP**	0.0101	0.4755*	0.1950	−0.2477	−0.0740	−0.0808	−0.2170	0.0312	0.0127	−0.1957	0.2535	0.3753*	
**Homycystein**	0.1560	−0.1703	−0.0211	−0.3107	−0.2449	−0.1346	0.0997	−0.1318	−0.1480	0.0075	0.1089	0.1273	
**LPA-2**	−0.2812	−0.1576	−0.0745	−0.0087	0.0515	−0.0607	−0.3077	−0.1058	0.1099	−0.3918*	−0.0862	−0.1950	
**Vitamin B12**	0.0312	−0.0769	−0.0495	−0.2951	−0.4599	−0.2833	−0.1180	−0.3590	−0.0262	0.0185	−0.1438	−0.1941	

Multivariable analysis of miR levels and certain surrogate markers of CHD. Spearman's rank correlation coefficient are given with asterisks indicating significant positive or negative correlations set by a p-value <0.05 (*).

**Table 4:** Associations between coronary sinus miR levels and surrogate markers of cardiovascular disease.

Marker	miR-17	miR-21	miR-34	miR-92	miR-126	miR-133	miR-145	miR-155	miR-181	miR-208	miR-221	miR-222	
**Hemoglobin**	0.0677	−0.0368	0.3365*	−0.1227	0.4724*	−0.0348	−0.1850	−0.1890	0.3315	0.2128	0.2536	0.1602	
**Leukocytes**	0.0816	−0.1025	−0.1758	0.1375	0.2642	0.2073	0.2831	0.2733	−0.1854	−0.0499	−0.0165	0.0684	
**Monocytes**	−0.0161	−0.1015	0.1631	−0.0413	0.2811	0.1336	0.4713*	0.2803	0.2680	−0.1607	0.1521	0.0479	
**Lymphocytes**	0.1357	−0.1751	0.3022	0.0822	0.0159	0.1901	0.2319	0.1018	0.2826	0.0161	0.2680	0.0745	
**Platelets**	−0.2285	−0.0986	0.0920	−0.3743*	−0.0642	0.2422	0.3232	0.0904	0.1349	0.0259	0.1440	0.1812	
**Total Cholesterol**	−0.0598	0.0532	0.0455	0.0300	−0.0239	0.1806	−0.0209	−0.0086	−0.0553	0.2603	0.0023	−0.0136	
**LDL-Cholesterol**	−0.0712	0.0198	−0.0552	−0.0071	0.0630	0.2030	0.0257	0.1013	−0.1141	0.2471	−0.0464	−0.0157	
**HDL-Cholesterol**	−0.2169	0.1301	0.1685	−0.0701	−0.1420	−0.2494	−0.1602	−0.2434	0.1732	0.0352	0.2511	0.2300	
**Triglycerides**	0.1909	−0.0065	0.1381	−0.0816	0.0983	0.3272	−0.1362	0.0813	−0.1870	0.1150	−0.1989	−0.1705	
**Glucose**	−0.0353	0.0052	−0.3173	0.1435	0.2346	0.3712*	0.0260	0.1365	−0.3721*	−0.1724	−0.1853	−0.2754	
**HbA1c**	−0.1202	−0.3273	0.0332	−0.1576	0.0122	0.0404	0.2455	0.1549	0.0310	−0.0434	0.0483	−0.0071	
**Insulin**	−0.0032	0.1207	−0.2042	0.1294	−0.0506	0.1030	−0.0371	−0.0883	−0.3219	−0.1858	0.0053	0.1492	
**BNP**	0.1442	0.0051	0.0647	0.2027	0.1747	0.0616	0.0941	0.0519	−0.0952	−0.0277	−0.0774	−0.1399	
**hs-CRP**	−0.1655	−0.0192	−0.1665	0.0119	0.0060	−0.0499	0.1987	0.0485	−0.0896	−0.3033	0.0478	0.2048	
**Homycystein**	−0.0817	0.1460	0.0064	−0.2101	−0.0618	−0.1340	0.1215	−0.1450	0.1662	−0.0978	0.1951	0.0630	
**LPA-2**	−0.0724	−0.4202*	0.1536	−0.2022	−0.0401	−0.1479	−0.1124	−0.0020	−0.0354	−0.2309*	−0.0538	−0.2161	
**Vitamin B12**	0.1683	−0.0283	0.3259	−0.0721	−0.2911	−0.1933	−0.3486*	−0.1650	0.0784	−0.1978	−0.2482	−0.1628	

Multivariable analysis of miR levels and certain surrogate markers of CHD. Spearman's rank correlation coefficient are given with asterisks indicating significant positive or negative correlations set by a p-value <0.05 (*).

**Table 5:** Associations between miR transcoronary gradients and surrogate markers of cardiovascular disease.

Marker	miR-17	miR-21	miR-34	miR-92	miR-126	miR-133	miR-145	miR-155	miR-181	miR-208	miR-221	miR-222	
**Hemoglobin**	0.0546	0.1928	0.4649*	−0.1441	−0.1415	0.0922	−0.0865	0.0566	0.4106*	0.2614	0.4083*	0.3984*	
**Leukocytes**	−0.0017	−0.3519*	−0.1621	0.1603	0.1307	−0.0774	−0.0318	0.0344	−0.2030	−0.1092	−0.1142	0.1009	
**Monocytes**	0.0765	−0.2119	−0.0123	0.1150	0.0298	−0.2199	0.2180	0.1163	0.0361	−0.0144	−0.0395	−0.1186	
**Lymphocytes**	0.0640	−0.2106	0.0515	0.0091	−0.1566	−0.3270	0.1130	−0.1432	0.2354	−0.0807	0.1601	0.0130	
**Platelets**	−0.3451*	−0.4609*	−0.4521*	0.0303	0.0999	0.2495	0.2413	0.1287	−0.2106	−0.0994	−0.3931*	−0.3208	
**Total Cholesterol**	−0.1368	−0.0465	−0.0911	−0.4362*	−0.1271	−0.3029	−0.2273	0.0038	−0.2654	−0.1476	−0.1195	0.0005	
**LDL-Cholesterol**	−0.1325	−0.1507	−0.2527	−0.3548*	0.0008	−0.2394	−0.1137	−0.1039	−0.4494*	−0.1856	−0.2939	−0.0730	
**HDL-Cholesterol**	−0.3150	0.1843	0.0262	−0.2019	−0.0526	−0.3273	−0.2314	−0.2618	0.0381	−0.0923	0.2418	0.1641	
**Triglycerides**	0.1099	0.0695	0.2406	−0.2693	−0.2455	−0.0425	−0.1181	0.1376	−0.1148	−0.0906	−0.1392	−0.0997	
**Glucose**	−0.0505	−0.1132	−0.2931	−0.0354	0.1145	0.1595	−0.0496	0.0994	−0.4510*	−0.1724	−0.2753	−0.2236	
**HbA1c**	−0.0570	−0.3941*	−0.3017	0.1322	0.0480	−0.0045	0.1733	−0.0070	−0.0717	0.1429	−0.1788	−0.2436	
**Insulin**	0.1604	−0.3026	−0.2712	0.2870	0.0911	−0.0482	0.1573	−0.0153	−0.1996	−0.2463	−0.1320	0.1507	
**BNP**	0.1624	0.2288	0.2766	0.2180	0.1577	0.1385	0.0859	0.1123	0.1758	0.1275	0.1991	−0.0941	
**hs-CRP**	−0.0018	−0.5302*	−0.3590	0.3245	0.1202	0.0959	0.4359*	−0.0726	−0.2518	−0.0274	−0.2717	−0.2133	
**Homycystein**	−0.0991	0.3266	0.0139	0.0821	0.0199	−0.2247	0.0686	0.0843	0.2869	−0.1538	0.1891	−0.1466	
**LPA-2**	0.2182	−0.2258	0.0483	−0.2206	−0.0522	0.0614	0.1981	0.0664	−0.1326	0.2922	−0.0039	−0.0695	
**Vitamin B12**	0.0312	0.0746	0.2665	−0.2951	−0.4599*	−0.2833	−0.1180	−0.3590*	−0.0246	0.0185	−0.0322	0.0655	

Multivariable analysis of miR levels and certain surrogate markers of CHD. Spearman's rank correlation coefficient are given with asterisks indicating significant positive or negative correlations set by a p-value <0.05 (*).

## Discussion

The current studies demonstrate that patients with early coronary atherosclerosis manifested by coronary endothelial dysfunction (CED) are characterized by, predominantly, elevated transcoronary gradients of endothelial and myocardial miR levels (miR-92a and miR-133, respectively). The levels of specific miRs were elevated in the coronary sinus compared to the left main coronary artery indicating a net release of the miRs within the coronary circulation. Furthermore, transcoronary gradients are evident early in atherosclerotic coronary disease, and with correlations of certain miRs with surrogate markers of CVD such as total cholesterol, LDL-cholesterol, triglycerides, vitamin B12, and hs-CRP. Based on these data circulating levels miR-92a and miR-133 could serve as surrogate markers for early coronary atherosclerosis. Thus, endothelial, myocardial, inflammatory, and smooth muscle related miRs might play a role within the coronary circulation in CED, an early stage of overt atherosclerotic coronary disease.

Our results provide an extension to previous data which show elevated transcoronary gradients of certain miRs in the setting of ACS [22], [23], yet advances this notion by demonstrating that the net release across the coronary circulation of these post-translational modifiers of endothelial function and myocardial remodeling occur early in the disease process. The presence of near ubiquitous elevations on transcoronary miR gradients indicates a production of such genomic suppressors associated with CED within the coronary circulation and may contribute the progression and complications of coronary artery disease.

Notably, these data show a significantly positive transcoronary gradient for miR-92a, indicating its net release from the coronary circulation (Figure 1c). Prior work in stable CHD has shown a reduction in miR-92a levels compared to controls [22], and even upregulation in miR-92a in the systemic circulation, resulting in a negative transcoronary gradient, in the setting of ACS [23]. On the other hand, previous *in-vitro* studies demonstrated that inhibiting miR-92a tends to improve endothelial function and repair [38] as well as angiogenesis and endothelial cell migration [8], [39]. Our current study is in accord with these latter observations, and a significantly elevated transcoronary gradient of miR-92a may indicate a release into the coronary circulation either causing, or as a result of, endothelial damage. Thus, our *in vivo* data from a population with early CHD offers an interesting glimpse into the transitional stage of CED between normal endothelial function and CHD, as well as a probable role for miR-92a in the progression, complications and potential therapeutic target for coronary artery disease.

The other miR that showed a significantly elevated gradient, miR-133, is thought to be protective and anti-fibrotic in smooth muscle [14] and is elevated in patients with coronary atherosclerosis [22]. Similar to our results, a study in patients with ACS has found a positive transcoronary gradient of miR-133 [18]. Our current study, furthermore, demonstrates a correlation between transcoronary gradient of miR-133 and the percent change in CBF with implications for myocardial ischemia dynamics. It may be speculated that in patients with myocardial injury and continual low-grade ischemia, there is a compensatory anti-fibrotic and myocardial protective process ongoing as the overabundance of miR-133 spills into the coronary circulation; alternatively miR-133 may serve a signal to the anti-ischemic process [40] secondary to the impaired tissue perfusion. These changes have been thought to occur through either a hypoxia-induced MAPK pathway [14] or possibly an anti-apoptotic, Bcl-2/Bax pathway which is initiated by inhibiting Caspase-9 directly [40]. The current study emphasizes the importance of identifying and treating early coronary disease to prevent myocardial damage and further dysfunction.

Controversy exists regarding whether the miR levels are altered or stay relatively stable with CHD. While Fichtlscherer et al. initially showed significant reductions in most absolute and relative endothelial-related miR levels (miR-17, miR-92a, and miR-126) with increases in myocardial and inflammatory-related miRs (miR-133, miR-145, and miR-155) in patients with stable CHD [22], De Rosa et al. showed little to no change in miR levels with stable CAD, but marked increases and decreases in some miR levels with ACS [23]. This group also showed increased transcoronary gradients in patients experiencing active ACS in muscle-enriched miRs, but not in vascular and cell-mediated miRs only. Our observation of a trend toward ubiquitous increases in miR transcoronary gradients in patients with early atherosclerosis is likely due to the stable nature of the participants very early in the disease process. Thus, it is likely that the positive transcoronary gradient of endothelial and anti-fibrotic miRs (miR-92a and miR-133), which may potentially become negative during ACS [23], could reflect a protective retention during ACS. Consequently, these elevated transcoronary gradients are present in patients without overt CHD but with endothelial dysfunction likely owing to chronic, ongoing inflammation and tissue damage.

Mechanistically, it is hard to delineate the temporal evolution of miRs from normal patients to those with microvascular CED, macrovascular CED, or even frank CHD as they change throughout the disease process. Likely, specific miRs have a protective effect on the coronary endothelium, and their reduction secondary to environmental stressors such as hypoxia, inflammatory mediators, or reactive oxygen species could lead to the development of atheromatous plaques [41]. This work also shows significant correlations between certain miR levels in both aortic and CVS samples as well as transcoronary gradients with surrogate markers of CHD such as monocytes, lymphocytes, platelets, lipids, glucose, LP(a), and hsCRP (Tables 3−5). These data show that certain surrogate markers of CHD – typically associated with deleterious changes in CED – are correlated with miRs in patients with a relatively low Framingham Risk Score (median  = 2%). Significant associations between miR-92a and total as well as LDL-cholesterol point to a logical correlation between substances known to be harmful to the endothelium (lipids) and miRs thought to be deleterious to endothelial health. A positive association between the miR-145 gradient and hsCRP coupled with the positive association between monocytes and miR-145 CVS levels could signal an ongoing cell-mediated inflammatory process within the vascular smooth muscle of the coronary circulation. While confounders can easily mar miR research, our results indicate that there were no statistical differences in medication profiles between the two different endothelial function groups. We also found no differences in the results when groups were stratified by aspirin use – a common potential confounder [42]. Moreover, the samples were all handled in a similar fashion reducing the room for confounders associated with laboratory protocols. These data, and others, point to an ongoing inflammatory process within the coronary circulation clearly involving these miRs in the process in this relatively low-risk population. Interestingly, there was a significant reduction in vitamin B12 levels in those with endothelial dysfunction (Table 2), however adjustment for these data made no impact on the overall results. While these findings might be hypothesis generating, there is no established biologic mechanism which could plausibly explain these finding. Nevertheless, these inflammatory processes and concomitantly dynamic miRs provides a potential link between the primary known consequence of vascular inflammation – endothelial dysfunction [33].

In summary, we report significantly elevated transcoronary gradients of miR-92a and miR-133 in patients without early coronary atherosclerosis and demonstrated coronary microvascular endothelial dysfunction. These results indicate a production of such miRs within the coronary circulation, either as a passive byproduct or an active contributor to such a process. This work establishes the role for alterations in miR profiles in those with early atherosclerosis and endothelial dysfunction at high risk for future CHD and the consequences thereof.
